# ^18^F-Fluorodeoxyglucose–Positron Emission Tomography/Computed Tomography Guided Stereotactic Body Radiation Therapy in Advanced Breast Cancer Patients Treated WithCyclin-Dependent Kinase 4/6 Inhibitors

**DOI:** 10.1016/j.adro.2026.102052

**Published:** 2026-04-07

**Authors:** Marcin Kubeczko, Berta Sousa, Francisco Oliveira, Cláudia Constantino, Catarina Duarte, Joana Castanheira, Leonor Matos, Justyna Kociołek, Michał Jarząb, Durval Costa, Fatima Cardoso, Javier Morales, Carlo Greco, Ana Luisa Vasconcelos

**Affiliations:** a Breast Cancer Center, Maria Sklodowska-Curie National Research Institute of Oncology, Gliwice Branch, Gliwice, Upper Silesia, Poland; b Breast Unit, Champalimaud Clinical Centre/Champalimaud Foundation, Lisbon, Portugal; c Nuclear Medicine-Radiopharmacology, Champalimaud Clinical Centre/Champalimaud Foundation, Lisbon, Portugal; d Radiotherapy Service, Champalimaud Clinical Centre/Champalimaud Foundation, Lisbon, Portugal; e ABC Global Alliance, Lisbon, Portugal

## Abstract

**Purpose:**

Cyclin-dependent kinase (CDK) 4/6 inhibitors have transformed the treatment of hormone receptor–positive/human epidermal growth factor receptor 2–negative metastatic breast cancer, yet resistance remains common. Metabolic response assessed by fluorine 18–labeled deoxyglucose might identify patients at risk of treatment failure earlier than conventional imaging. We evaluated the safety, feasibility, and efficacy of ^18^F-fluorodeoxyglucose–positron emission tomography/computed tomography (^18^F-FDG-PET/CT)–guided stereotactic body radiation therapy(SBRT) combined with CDK4/6 inhibitors.

**Methods and Materials:**

This retrospective study included 29 patients monitored with ^18^F-FDG-PET/CT, who received SBRT concurrently with CDK4/6 inhibitors treatment or before CDK4/6 inhibitors commencement. Median age was 61 years (IQR, 53-68). Ten patients had de novo disease. The median disease-free interval for patients with recurrent disease was 66.9 months (IQR, 34.5-108.5). The majority of patients (72%) were treated in the first-line setting; 11 received ribociclib, 15 received palbociclib, and 3 received abemaciclib. Fifty-nine radiation treatments were delivered to 70 lesions: 41 before or concomitantly with the first CDK4/6 inhibitors cycle and 18 for oligoprogressive disease. Twenty-six treatments used single-fraction SBRT, and 33 used hypofractionated SBRT.

**Results:**

Post-SBRT maximum standardized uptake value (SUV_max_) reduction was observed in all but one lesion. Metabolic complete response (mCR) occurred in 33 sites (56%), more frequently after single-fraction SBRT than hypofractionated SBRT (*P* = .03), in smaller lesions (<14 cm^3^, *P* = .002), and in lesions with lower SUV_max_ (<6.3, *P* = .008). Each unit increase in SUV_max_ reduced odds of mCR by ∼18% (odds ratio, 0.824; *P* = .04). Median progression-free survival was 48.2 months; 2-year progression-free survival was 71.5% (95% confidence interval [CI], 50.9-84.6).

**Conclusions:**

^18^F-FDG-PET/CT–guided SBRT, particularly single-fraction, added to CDK 4/6 inhibitors, is a valuable treatment modality resulting in substantial rates of mCR. Whether this translates into deferring disease progression requires randomized studies with larger treatment groups. To our knowledge, this study represents the first analysis evaluating ^18^F-FDG-PET/CT–guided SBRT in combination with CDK4/6 inhibitor–based therapy in patients with metastatic breast cancer.

## Introduction

Breast cancer is the most commonly diagnosed cancer globally.^1^ Cyclin-dependent kinase (CDK) 4/6 inhibitors play a crucial role in the treatment of hormone receptor–positive (HR+)/human epidermal growth factor receptor 2–negative (HER2-negative) metastatic breast cancer (MBC), improving progression-free survival (PFS), overall survival (OS), and quality of life (QoL).^2^ Nonetheless, intrinsic and acquired resistance are common, underscoring the need for predictive biomarkers; liquid biopsy is promising, but imaging remains central for response monitoring.^3^^,^^4^

^18^F-fluorodeoxyglucose–positron emission tomography/computed tomography (^18^F-FDG-PET/CT) quantifies tumor glycolytic activity,^5^ has been extensively studied in breast cancer,^6^ predicts pathologic response after neoadjuvant chemotherapy,^7^ and in the metastatic setting, sharpens response stratification.^8^ Metabolic response to CDK4/6 inhibitors correlates with prolonged PFS, and early ^18^F-FDG-PET/CT may identify patients at risk of treatment failure^9^^,^^10^; whether nonresponders benefit from adapted regimens is unknown.

Stereotactic body radiation therapy (SBRT) is an important modality for selected MBC patients, delivering ablative doses in one or few fractions while sparing normal tissues.^11^ Evidence on ^18^F-FDG-PET/CT response with SBRT plus targeted therapy is limited. The EORTC‑ESTRO OligoCare consortium issued consensus recommendations for combining metastasis‑directed SBRT with targeted or immunotherapy; however, given limited retrospective data for CDK4/6 inhibitors, no consensus on same‑day administration or minimal intervals was reached.^12^ An ESTRO‑endorsed international consensus did not recommend concomitant radiation therapy (RT)–CDK4/6 inhibitors in the adjuvant setting due to insufficient evidence, but supported case‑by‑case use in the metastatic setting.^13^

Single-fraction SBRT (SF-SBRT) may confer distinct radiobiology and high ablation rates.^14^ ^18^F-FDG-PET/CT–derived volumetric and metabolic metrics can guide radioablation and predict polymetastatic conversion after RT.^11^ Trials adding local therapy to systemic treatment have yielded mixed results: BR002 was negative for PFS/OS,^15^ whereas COMET suggested a survival benefit in selected patients,^16^ highlighting the need for careful selection.

Accordingly, we assessed the safety, feasibility, and efficacy of ^18^F-FDG-PET/CT–guided SBRT (including single-fraction) in advanced breast cancer treated with CDK4/6 inhibitors.

## Methods and Materials

### Study population

This retrospective study included patients treated with CDK4/6 inhibitors between January 2017 and June 2023 who were assessed for eligibility. The inclusion criteria were: (1) HR+/HER2-negative MBC; (2) scheduled for treatment with CDK4/6 inhibitors combined with hormone therapy (letrozole, fulvestrant, exemestane, tamoxifen or anastrozole); (3) breast cancer with cells avid for ^18^F-FDG before CDK4/6 inhibitors therapy (baseline); (4) SBRT performed either within 12 months prior to CDK4/6 inhibitor initiation or concurrently with CDK4/6 inhibitor treatment; (5) treatment efficacy monitored with ^18^F-FDG-PET/CT examinations, performed before and after RT. No predefined SUV cut-off was applied, as eligibility was based on the presence of visually ^18^F-FDG-avid disease suitable for response assessment on serial ^18^F-FDG-PET/CT examinations. The study was conducted in accordance with the Declaration of Helsinki and approved by the Ethics Committee of Champalimaud Clinical Center (approval no. 2025021802). Informed consent was obtained from all living patients. For deceased patients, a waiver of consent was granted by the ethics committee.

### CDK4/6 inhibitor treatment and timing of RT

Every CDK4/6 inhibitor cycle comprises 28 days. Ribociclib and palbociclib are taken for 21 days, followed by a 7-day treatment-free period, whereas abemaciclib is taken for 28 days without interruptions. The half-life of palbociclib is 28.8 hours (6 half-lives, 6 days),^17^ the half-life of ribociclib is 32.0 hours (5 half-lives, 6.7 days),^18^ and the half-life of abemaciclib is 24.8 hours (5 half-lives, 5.2 days).^19^

RT timing in relation to CDK4/6 inhibitor administration was not protocol-mandated. RT was delivered independently of CDK4/6 inhibitor cycle timing and was not systematically scheduled during treatment-free intervals. SBRT was delivered either during ongoing CDK4/6 inhibitor treatment or following temporary treatment interruption, depending on clinical judgment. The decision to continue or temporarily suspend CDK4/6 inhibitors during SBRT was left to the discretion of the treating physician. In some cases, CDK4/6 inhibitor administration was continued throughout SBRT, whereas in others, it was briefly suspended before SBRT initiation or after the first SBRT fraction.

For each patient, the timing of SBRT in relation to CDK4/6 inhibitor administration was individually and precisely documented, including treatment continuation or temporary suspension, as detailed in Table E1.

### SBRT techniques

^18^F-FDG-PET/CT was used for SBRT treatment planning, to outline the gross tumor volume, and to contour the clinical target volume (CTV) in some cases. A 3 mm margin was added to the gross tumor volume or CTV to generate the treatment planning target volume (PTV). Treatment planning was performed with Eclipse software (Varian Medical Systems) using volumetric modulated arc therapy. The same treatment planning guidelines were used for SF-SBRT and hypofractionated SBRT (HF-SBRT). The EDGE platform (Varian Medical Systems), equipped with an optical surface monitoring system and an ExactCouch system for a 6-degrees-of-freedom patient setup, was used for treatment delivery. Patients received total doses ranging from 16 to 36 Gy, administered either as single-fraction treatment (16-24 Gy) or as hypofractionated regimens (eg, 25 Gy in 5 fractions, 30 Gy in 6 fractions, 35 Gy in 7 fractions, or 36 Gy in 12 fractions), tailored according to tumor location, size, and clinical considerations.

### Endpoints

Definitions of tumor response were based on the EORTC guidelines,^20^ using ^18^F-FDG-PET/CT scans performed before and after SBRT. The median interval between baseline ^18^F-FDG-PET/CT assessment and initiation of SBRT was 1.0 months. The median interval between SBRT completion and subsequent ^18^F-FDG-PET/CT response assessment was 3.9 months. Metabolic complete response (mCR) was defined as a complete resolution of ^18^F-FDG uptake in the irradiated lesion. We accepted a level of ≥25% reduction in the maximum standardized uptake value (SUV_max_) as a metabolic partial response, as was done in previous studies.^9^^,^^21^ Progressive metabolic disease was defined as a >25% increase in SUV_max_ or the development of a new ^18^F-FDG-avid lesion, whereas an increase or decrease of <25% was a stable metabolic disease.

The duration of local control (LC) was calculated for each lesion site independently from the date of radioablation completion. The duration of PFS was calculated for each patient from the CDK4/6 inhibitors commencement date, whereas OS was calculated from the date of metastatic disease diagnosis. Acute and late toxicities were scored based on the National Institutes of Health National Cancer Institute Common Terminology Criteria for Adverse Events Guidelines, version 5.0. High-grade nonhematologic adverse events were also assessed with toxicity criteria of the Radiation Therapy Oncology Group and the EORTC.

The primary objective was to evaluate the safety, feasibility, and efficacy of ^18^F-FDG-PET/CT–guided SBRT (including single-fraction) in advanced breast cancer patients treated with CDK4/6 inhibitors. The secondary objective was to assess the efficacy of SBRT in the subpopulation of patients with oligoprogressive disease (OPD).

### Feasibility of ^18^F-FDG-PET/CT monitoring and ^18^F-FDG-PET–guided SBRT with CDK4/6 inhibitors treatment

At our institution, ^18^F-FDG-PET/CT monitoring and ^18^F-FDG-PET–guided SBRT during CDK4/6 inhibitor treatment were successfully implemented as part of routine clinical care. All patients underwent baseline ^18^F-FDG-PET/CT, and longitudinal ^18^F-FDG-PET–based assessments were available throughout the course of treatment. This enabled lesion-specific monitoring and facilitated informed decisions regarding local therapy. SBRT was applied both prior to or at the initiation of CDK4/6 inhibitors treatment in selected lesions and as a response-adapted strategy for OPD, based on the differential metabolic response across lesions. No technical or logistical barriers to implementation were encountered in our high-volume center with established ^18^F-FDG-PET/CT imaging and RT workflows.

### Statistical analysis

Categorical variables were shown as frequencies and percentages. Pairwise comparisons between patient subgroups were performed by Fisher's exact test. Continuous data were summarized as median values with interquartile ranges (IQRs, 25% to 75%). PFS, OS, and LC were estimated using the Kaplan-Meier method, and 95% confidence intervals (CIs) for the survival curves were calculated. PFS was calculated from initiation of CDK4/6 inhibitor therapy. Median PFS was derived from Kaplan-Meier estimates and could be calculated despite a shorter median follow-up, as the survival curve crossed the 50% threshold during the observation period. Differences between variables were assessed with the Wilcoxon rank sum test. Receiver operating characteristic analysis was used to assess optimal cut-off values. Logistic regression was performed to build a model for mCR prediction. A *P* value <.05 was considered statistically significant. The statistical tests were conducted with Stata Statistical software (version 18, StataCorp).

## Results

### Study population

One hundred forty-nine consecutive patients with HR+/HER2-negative MBC treated with CDK4/6 inhibitors were analyzed. Twenty-nine patients, monitored with ^18^F-FDG-PET/CT, received SBRT concurrently with CDK4/6 inhibitors treatment or within 12 months of CDK4/6 inhibitors commencement and were included in the study. The median age was 61 years (IQR, 53-68). Ten patients had de novo disease, whereas 19 received diagnoses of recurrent disease. Bone metastases were present in 24 patients (83%), representing the most common metastatic site. The median disease-free interval in patients with recurrent disease was 66.9 months (IQR, 34.5-108.5). The majority of patients were treated in the first-line setting (21, 72%), 5 in the second-line, 2 in the third-line, and 1 in the fifth line. Eleven patients received ribociclib, 15 received palbociclib, and 3 received abemaciclib. As an endocrine compound, 18 patients were on letrozole, 10 on fulvestrant, and 1 on exemestane. Eleven patients received chemotherapy previously in the neo/adjuvant setting. The majority of patients with bone metastases received denosumab as a bone-modifying agent.

### SBRT details

Fifty-nine treatments for 70 lesions were performed. In most cases, SBRT was performed due to oligoprogressive or oligometastatic disease. Twenty-eight SBRT treatments were performed before CDK4/6 inhibitors commencement with a median time of 14 days between SBRT and CDK4/6 inhibitors (IQR, 8-17 days). Treatment details are shown in Table 1. Thirteen SBRT treatments were performed at the beginning of CDK4/6 inhibitor treatment, concurrently with the first CDK4/6 inhibitor cycle, whereas 18 treatments were performed during subsequent CDK4/6 inhibitor cycles due to OPD. In total, 31 SBRT treatments were performed concurrently with CDK4/6 inhibitors (52.5%), whereas 28 SBRT treatments were completed before CDK4/6 inhibitors commencement. Twenty-six treatments for 32 lesions were performed with SF-SBRT. Details on the irradiated volumes are provided in Table E2.

**Table 1:** Treatment details of patients irradiated before CDK4/6 inhibitor commencement or during the first cycle

Sequential SBRT							
Pt No.	Age (y)	Treated site	SUV_max_ before	Dt/Dfx	SUV_max_ after	LC (mo)	RTint (d)
1	57	Sacrum + ilium Rt	5.6	25/5	4.5	33.9	6
2	54	T6	5.3	24/24	mCR	36.8	10
2	54	Ilium Rt + Lt (4 lesions)	4.0	16/16	mCR	36.8	10
2	54	Scapula Lt	3.5	24/24	mCR	36.9	13
3	58	Supraclavicular LNs Lt	3.3	35/7	mCR	52.9	6
4	68	C2	8.0	25/5	mCR	42.3	14
4	68	L3	8.3	25/5	3.1	42.3	14
4	68	L5	11.0	25/5	mCR	42.3	14
5	68	Ischium Lt	5.4	24/24	mCR	14.2	182
5	68	Sacrum	5.3	21/21	mCR	14.2	182
6	86	Ilium Rt + sacrum	14.7	25/5	5.5	22.7	10
7	75	Mediastinal LNs (2 lesions)	10.0	21/21	5	42.3	184
8	64	Sternum	15.0	35/7	4	41.4	2
9	48	Humerus Rt	4.1	21/21	mCR	34	267
10	57	Ilium Lt	7.0	25/5	mCR	18.7	2
12	63	C3	11.0	25/5	3	29.3	16
12	63	Ilium Rt	9.0	35/7	4.4	29.2	14
13	64	Femur Rt	6.0	30/6	mCR	16.9	14
19	48	L3	5.7	24/24	mCR	18.1	5
19	48	L4	3.5	24/24	mCR	18.1	5
20	85	L5	6.2	35/7	mCR	15.2	208
21	46	Lumbosacral	8.8	25/5	3	10.8	26
25	48	Internal mammary LNs (4 lesions)	7.0	35/7	2.9	11.1	11
25	48	Rib 9 Rt	6.5	24/24	3.2	11.4	17
26	52	T 5-9 + ribs 6-9	16.5	25/5	3	10.4	17
26	52	Ilium Rt	9.0	25/5	3	10.4	9
29	77	T12	6.6	18/18	mCR	76.5	9
29	77	L2	8.9	18/18	mCR	76.5	7

Concomitant SBRT							
No.	Age (y)	Treated site	SUV_max_ before	Dt/Dfx	SUV_max_ after	LC (mo)	CDK C1
1	57	T8-9	5.4	25/5	4.5	33.2	7
11	57	Femur Rt	8.9	25/5	mCR	12.4	19
11	57	Sacrum + ilia (Rt+Lt)	17.6	25/5	3.8	12.4	14
14	61	T 6-8	7.0	25/5	5.6	26.5	14
14	61	Rib 10 Lt	13.0	25/5	10.2	26.5	4
16	48	T 9	8.0	24/24	mCR	25.3	28
16	48	Skull	13.0	22/22	mCR	25.3	28
17	74	Sacrum-ilium Rt	14.4	30/6	7.2	26.0	11
23	59	T11	6.5	35/7	3.7	50.0	17
23	59	Sacrum-ilium Lt	4.6	35/7	4.6	50.0	17
24	58	Sacrum	10.1	25/5	mCR	14.6	9
28	61	Rib 12 R	4.0	36/12	mCR	47.8	9
28	61	T7	6.8	36/12	mCR	47.8	7

*Abbreviations:* C = cervical spine; L = lumbar spine; ; CDK = cyclin-dependent kinase; CDKC1 = day in the first CDK4/6 inhibitor cycle, when SBRT began; Dt/Dfx = total dose/dose per fraction; LC = local control; LN = lymph node; mCR = metabolic complete response; Pt No. = patient number; Lt = left; Rt = right; RTint(d) = interval between SBRT completion and CDK4/6 inhibitors commencement (days); SBRT = stereotactic body radiation therapy; SUV_max_ = maximum standardized uptake value; T = thoracic spine.

### OPD

**Figure 1:**
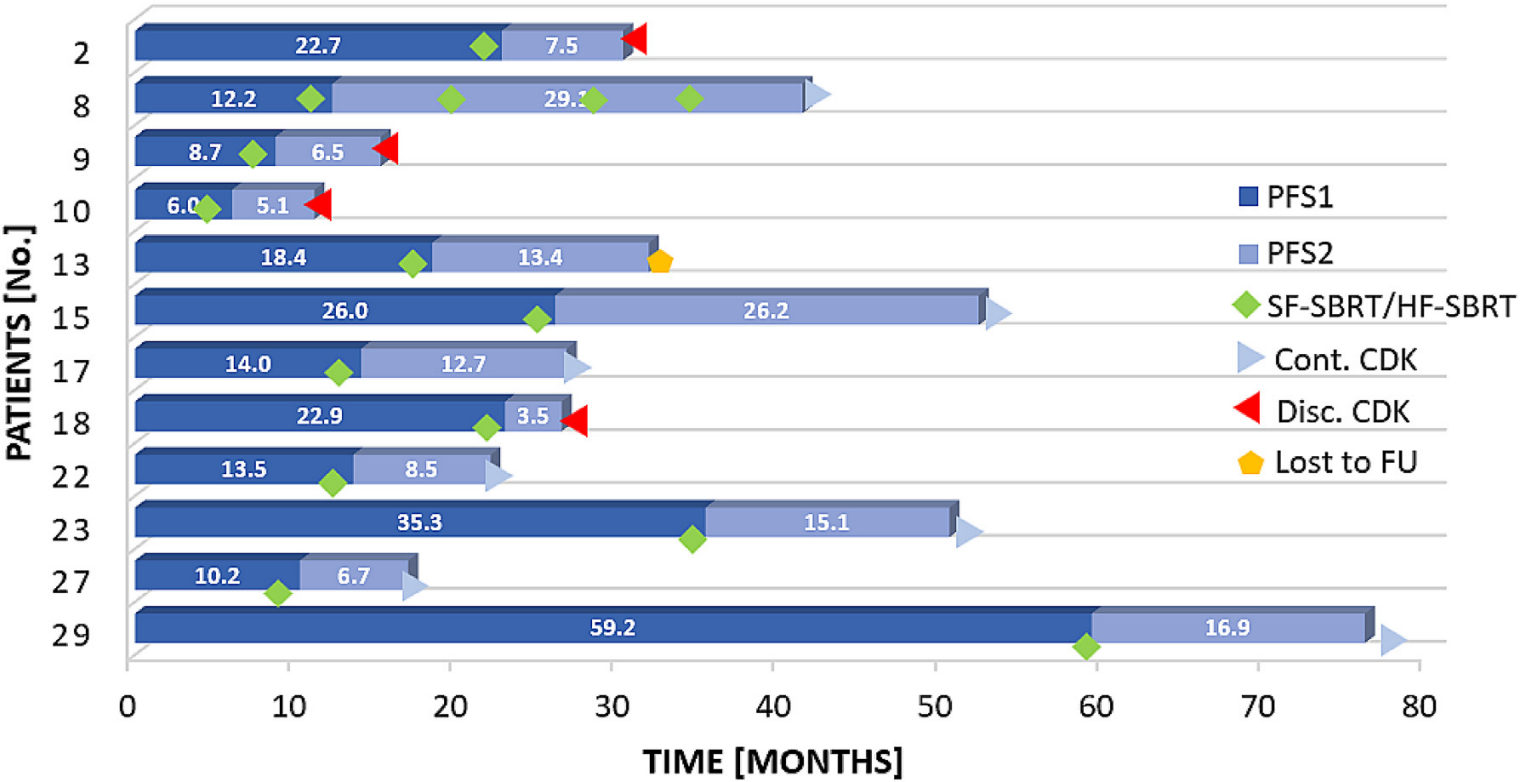
Outcomes in patients treated with SBRT for oligoprogression. *Abbreviations:* CDK = cyclin-dependent kinase 4/6 inhibitors; Cont. = continuation; Disc. = discontinuation; FU = follow-up; HF-SBRT = hypofractionated stereotactic body radiation therapy; PFS = progression-free survival; SF-SBRT = single-fraction stereotactic body radiation therapy. PFS1 was defined as the time from CDK4/6 inhibitor initiation to the occurrence of oligoprogression. PFS2 was defined as the time from the completion of SBRT for the oligoprogressive lesion to subsequent disease progression or last follow-up, whichever occurred first.

Patients with OPD amenable to SBRT were qualified for local treatment and CDK4/6 inhibitors continuation. Twelve patients had OPD during CDK4/6 inhibitor treatment after various periods of time. Eighteen sites were irradiated due to OPD (23 lesions). Six sites (9 lesions) were irradiated with HF-SBRT, whereas 12 sites (14 lesions) were treated with SF-SBRT. Of the patients, 83% remained without progression for ≥6 months. At the time of data cut-off, all lesions treated with SF-SBRT were controlled. Four patients had disease progression outside the irradiated area and were switched to another systemic treatment, and 1 patient was lost to follow-up. The remaining 7 patients continued treatment with CDK4/6 inhibitors. Treatment details are shown in Table 2 and Fig. 1.

**Table 2:** Treatment details of patients irradiated due to oligoprogression on CDK4/6 inhibitors

Pt No.	Age (y)	Characterization of OPD	OPD site	SUV_max_ before	Dt/ Dfx	SUV_max_ after	LC (mo)
2	54	De novo bone lesion in CT with ^18^F-FDG uptake	Ilium Lt (3 lesions)	3.6	30/6	2.6 mPR	13.6
2	54	Increase in ^18^F-FDG uptake (from 4.5 to 9.1)	L5	9.1	30/6	4.5 mPR	13.6
8	64	De novo bone lesion in CT with ^18^F-FDG uptake of 6.7, previously 4.3, not seen at baseline ^18^F-FDG-PET/CT	Sacrum	6.7	24/24	mCR	29.1
8	64	De novo bone lesion in CT with ^18^F-FDG uptake	T11	5.8	35/7	mCR	18.7
8	64	De novo bone lesion in CT with ^18^F-FDG uptake	Ilium Lt	5.6	24/24	mCR	7.2
9	48	Enlargement of LN from 5 mm to 11 mm, previously without ^18^F-FDG uptake	Mediastinal LNs (2 lesions)	4.4	35/7	mCR	16.3
10	57	Increase in ^18^F-FDG uptake (from 7.0 to 8.0)	L1	8.0	20/20	mCR	12.6
10	57	Increase in ^18^F-FDG uptake (from 5.0 to 6.0)	T4	6.0	20/20	mCR	12.6
13	64	Increase in ^18^F-FDG uptake (from 9.8 to 11.2)	T8	11.2	16/16	mCR	13.4
13	64	Increase in ^18^F-FDG uptake (from 4.6 to 5.8)	Ilium Rt	5.8	16/16	mCR	13.4
15	54	Increase in ^18^F-FDG uptake (from 7.5 to 14.4)	Liver metastasis	14.4	45/15	mCR	26.2
17	74	De novo bone lesion in CT with ^18^F-FDG uptake	L4	12.8	14/14	5.3 mPR	12.7
18	40	Increase in ^18^F-FDG uptake (from 3.8 to 6.3)	L4	6.3	21/21	3.3 mPR	18.5
22	83	De novo ^18^F-FDG uptake, no morphologic lesion in CT	T11	6.8	24/24	mCR	8.5
23	59	De novo bone lesion in CT with ^18^F-FDG uptake	T6	5.6	35/7	mCR	15.1
27	66	Increase in ^18^F-FDG uptake (from 4.0 to 6.5)	L5	6.5	24/24	3.6 mPR	6.7
27	66	Increase in ^18^F-FDG uptake (from 4.0 to 6.6)	Ilium Lt	6.6	24/24	2.9 mPR	6.7
29	77	Increase in ^18^F-FDG uptake (from <3 to 4.7; in the Bsl PET SUV_max_ 8.0)	Sacrum + ilium Rt (3 lesions)	4.7	16/16	2.8 mPR	16.9

*Abbreviations:* Bsl = baseline; CDK = cyclin-dependent kinase; CT = computed tomography; Dt/Dfx = total dose/dose per fraction; FDG = fluorodeoxyglucose; Lt = left; L = lumbar spine; LC = local control; LN = lymph node; mCR = metabolic complete response; mPR = metabolic partial remission; OPD = oligoprogressive disease; PET = positron emission tomography; Pt No. = patient number; Rt = right; SUV_max_ = maximum standardized uptake value; T = thoracic spine.

### Treatment efficacy

**Figure 2:**
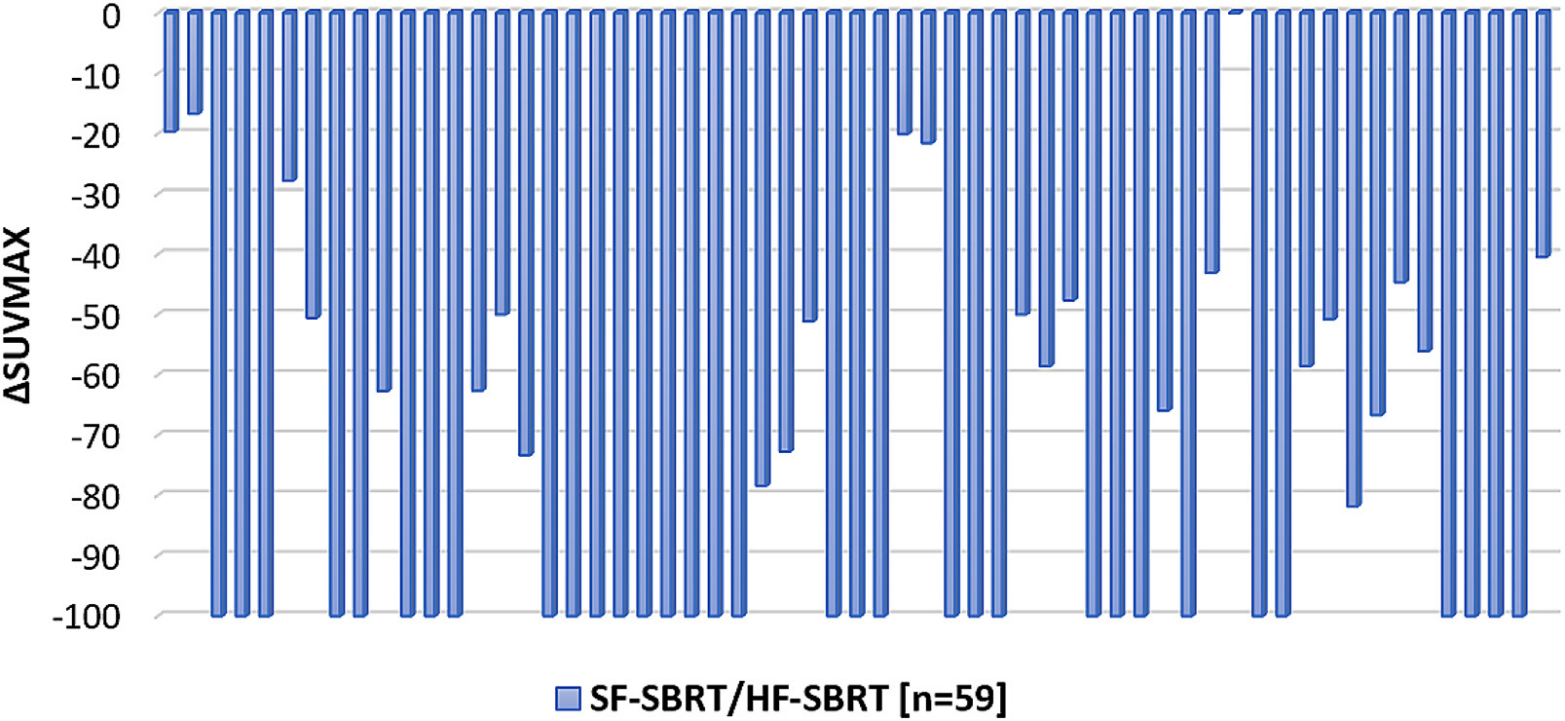
Percentage change in maximum standardized uptake value (SUV_max_) in irradiated sites after stereotactic body radiation therapy (SBRT). *Abbreviations*: HF-SBRT = hypofractionated stereotactic body radiation therapy; SF-SBRT = single-fraction stereotactic body radiation therapy.

A decrease in SUV_max_ was observed in all but one site after SBRT, and the results are depicted in Fig. 2. mCR was observed in 33 sites (56%). mCR after SBRT was significantly more frequent after SF-SBRT (19/26, 73.1%) compared to HF-SBRT (14/33, 42.4%; *P* = .03). Additionally, the mCR rate was significantly higher in smaller lesions (CTV < 14.14 cm^3^; 24/32, 75%), than in larger ones (CTV ≥ 14.14 cm^3^; 9/27, 33.3%; *P* = .002). mCR was significantly more frequent in lesions with baseline SUV_max_ <6.3 (18/23, 78.3%) compared to SUV_max_ ≥6.3 (15/36, 41.7%; *P* = .008). In logistic regression analysis, the baseline SUV_max_ value was significantly associated with achieving mCR (odds ratio = 0.824, *P* = .04), indicating that each unit increase in baseline SUV_max_ was associated with 18% decrease in the odds of achieving mCR after SBRT. The only site without any metabolic response (no decrease in SUV_max_) was a bone lesion located in the sacroiliac region (CTV = 115 cm^3^), treated with 35 Gy delivered in 5 fractions.

**Figure 3:**
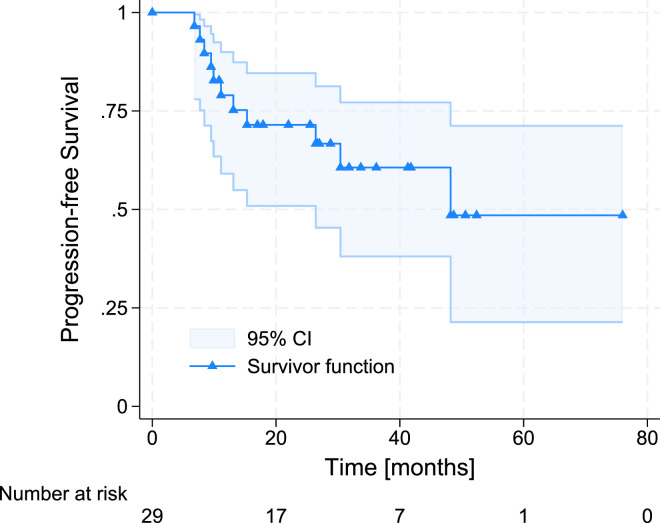
Treatment efficacy—progression-free survival.

The median follow-up time was 27.2 months (IQR, 17.6-42.9). At the time of data cut-off, 17 patients were still treated with CDK4/6 inhibitors, and one was lost to follow-up. The median PFS was 48.2 months, and the 2-year PFS was 71.5% (95% CI, 50.9%-84.6%). The PFS results are shown in Fig. 3.

Median OS and LC were not reached. Three-year OS was 94.7% (95% CI, 68.1%-99.2%). Two-year LC was 94.7% (95% CI, 79.0%-98.7%). Progression was observed in only 2 irradiated sites. In both cases, mainly due to particularly large PTV (sacroiliac area, 595.1 cm^3^; Th 5-9 + ribs 6-9, 376.1 cm^3^), the total dose was 25 Gy delivered in 5 fractions.

Eleven patients had disease progression and were switched to subsequent lines of systemic treatment. The sites of disease progression were: new bone lesions, new liver lesions, brain metastasis, or progression in lymph nodes. In the line of treatment following CDK4/6 inhibitors, patients received everolimus combined with hormone therapy, capecitabine, vinorelbine, and fulvestrant monotherapy. Most patients had short responses. The median PFS of that subsequent systemic treatment was 6 months.

### Safety of SBRT and CDK4/6 inhibitor combination

The SBRT was well tolerated. Five cases of high-grade myelotoxicity were reported after SBRT (17%). In the first patient, G3 neutropenia was found on CDK4/6 inhibitor C1D14, 16 days after completing sequential HF-SBRT with 35 Gy in 5 fractions for the sternum (PTV volume, 120.3 cm^3^). C2D1 was postponed for 7 days. In the second, G3 neutropenia was seen on CDK4/6 inhibitor C2D1, 2 weeks after completing concomitant HF-SBRT during the first CDK4/6 inhibitor cycle; 25 Gy in 5 fractions was delivered simultaneously to the spine (Th 6-8; PTV volume, 177.7 cm^3^) and the rib (PTV volume, 39.8 cm^3^). C2D1 was postponed for 14 days. In third patient, G3 neutropenia and G2 mucositis were observed after SF-SBRT with 24 Gy delivered simultaneously to the spine (T9; PTV volume, 5.8 cm^3^) and the skull (PTV volume 14.9 cm^3^); however, because it was reported in C1D21 (at the beginning of week-off), palbociclib was not postponed, and the subsequent CDK4/6 inhibitor cycle started as planned without interruptions. In the fourth patient, G3 neutropenia was observed on CDK4/6 inhibitor C2D1, 1 week after completing concomitant HF-SBRT in the first cycle with 30 Gy delivered in 5 fractions to the sacroiliac area R (PTV volume, 133.2 cm^3^). C2D1 was postponed for 7 days. Only one episode of high-grade myelotoxicity was reported among patients treated due to OPD. Neutropenia G3 was observed 4 weeks after HF-SBRT; however, similar episodes of neutropenia were also observed before HF-SBRT. HF-SBRT with 35 Gy in 5 fractions was delivered to the spine (T6; PTV volume, 7.7 cm^3^). The subsequent CDK4/6 inhibitor cycle was postponed for 7 days. In one patient, on the day of SF-SBRT, neutropenia G3 was found: SF-SBRT with 16 Gy to the spine (T8) was delivered as previously planned, and the subsequent ribociclib cycle was started 4 days later when neutropenia improved to G2. One G4 late toxicity (according to the Radiation Therapy Oncology Group) was reported in a 64-year-old patient irradiated to the right femur 16.9 months earlier (PTV volume, 196.2 cm^3^). A right peritrochanteric fracture occurred after falling. En bloc resection of the femur head and reconstruction with a total hip prosthesis were performed; no cancer cells were identified in the pathology studies. No other severe acute, subacute, or late toxicity after SBRT was observed. Several patients had some mild or moderate adverse events, such as G1 odynophagia (in 2 patients), G2 upper respiratory tract infection (in 1 patient), G2 nausea and vomiting (in 1 patient), G2 dysphagia (in 1 patient), G1 lumbalgia (in 1 patient), and herpes zoster infection (in 1 patient, in the irradiated area, 2 weeks after SBRT completion).

## Discussion

CDK4/6 inhibitors are the standard of care in HR+/HER2-negative MBC because of the OS, PFS, and QoL benefit.^2^ Nonetheless, acquired resistance is common, and optimal strategies after progression remain a major unmet need.^22^ Whether treatment intensification during CDK4/6 inhibitors confers additional benefit is uncertain, underscoring the importance of individualized approaches. Herein, we present the safety, feasibility, and efficacy of ^18^F-FDG-PET/CT–guided SBRT in this setting.

The median PFS in our study was 48.2 months, with a 2-year PFS of 71.5%. With similar follow-up, the median PFS in the MONALEESA-2 study was 25.3 months.^23^ Selection bias might be the reason for such differences because patients with a limited number and size of metastases are candidates for high-dose RT delivered in a highly conformal manner rather than palliative RT regimens.^24^ However, more than 40% of patients in our study were treated due to OPD, which is associated with a greater risk of progression or death.^25^ Preclinical studies have indicated the radiosensitization effects of CDK4/6 inhibitors, both in vitro and in vivo.^26^ Thus, a synergistic effect of SBRT and CDK4/6 inhibitors is plausible and merits further studies.

We found that adding SF-SBRT to CDK4/6 inhibitors provided excellent LC without any local failures. Some patients required HF-SBRT due to normal organ interference, and the 2-year LC was 94.7%, consistent with the previously reported 93% LC at 1 year.^25^ As expected, increasing tumor diameter is associated with inferior LC; the few local failures occurred in especially large targets. Follow-up was mature (median 27.2 months), exceeding that of many SBRT series in oligometastatic disease.^25^

In our study, early mCR was observed in more than half of the treated sites (56%), particularly after SF-SBRT (73%). However, these estimates refer exclusively to complete responses identified on the first post-SBRT ^18^F-FDG-PET/CT. Given that mCRs may occur several months after RT, the overall proportion of complete responses following SBRT may be higher.

Importantly, the potential benefit of integrating SBRT with CDK4/6 inhibitor therapy was observed irrespective of the timing of SBRT delivery. In this study, SBRT was applied across multiple clinically relevant time points, including prior to initiation of CDK4/6 inhibitors, early during treatment (around the first treatment cycle), as well as later in the course of therapy in the setting of oligoprogression. Notably, even when oligoprogression occurred after prolonged exposure to CDK4/6 inhibitors—more than 2 or 3 years after treatment initiation—the use of SBRT was associated with LC of the progressing lesion and enabled continued effective systemic treatment through maintenance of CDK4/6 inhibitor therapy, without the need to change systemic treatment. These observations have important clinical implications for routine practice, suggesting that optimal local treatment with SBRT may help preserve ongoing clinical benefit from CDK4/6 inhibitor–based systemic therapy, which is generally well tolerated. However, an important limitation of the present study should be acknowledged. Because multiple lesions were treated in some patients, lesion-based analyses should be interpreted with caution, as outcomes may not be fully independent.

From a clinical perspective, the present findings support a conceptual framework in which ^18^F-FDG-PET/CT–guided SBRT may be integrated with CDK4/6 inhibitor–based therapy at multiple stages of disease management. In selected patients with limited metastatic burden at the time of CDK4/6 inhibitor initiation, ^18^F-FDG-PET/CT–guided SBRT may be considered as part of an early treatment intensification strategy to achieve optimal LC of metabolically active disease sites. In addition, during ongoing CDK4/6 inhibitor therapy, ^18^F-FDG-PET/CT–guided SBRT may be applied in the setting of oligoprogression to ablate progressing lesions and potentially allow continuation of otherwise effective systemic treatment. Although this study was not designed to define a prescriptive treatment algorithm, these observations highlight how metabolic imaging–guided local therapy could be incorporated into individualized treatment strategies to support prolonged disease control in routine clinical practice.

A previously published study comprising various cancer histologies, performed predominantly before CDK4/6 inhibitor approval, reported that a combination of <14.8 cm^3^ initial tumor load and <6.5 SUV_max_ defines a favorable polymetastases-free survival population.^11^ Our optimal cut-offs for mCR after SBRT were strikingly similar: initial tumor load <14.1 cm^3^ and baseline SUV_max_ <6.3. These concordant thresholds support ^18^F-FDG-PET/CT as a practical decision tool for identifying patients most likely to benefit from local ablation in MBC.

Beyond baseline selection, ^18^F-FDG-PET/CT may enhance longitudinal management. Several studies suggest that ^18^F-FDG-PET/CT outperforms conventional imaging—including CT and bone scintigraphy—for monitoring therapeutic response in MBC,^27^ and ^18^F-FDG-PET/CT–derived radiomic or lesional features beyond SUV_max_ can add a biological and prognostic signal.^28^^,^^29^ ^18^F-FDG-PET/CT can also detect progression earlier than contrast-enhanced CT, enabling timelier adaptation of treatment.^30^ In prostate cancer, ^18^F-fluorocholine–PET/CT has identified patients who derive substantial benefit from SBRT.^31^ Comparable evidence is lacking in HR+/HER2-negative MBC treated with CDK4/6 inhibitors; to our knowledge, our analysis is the first to assess ^18^F-FDG-PET/CT–driven SBRT delivery in this homogeneous clinical scenario. Notably, in selected cases, ^18^F-FDG-PET/CT–guided SBRT to OPD sites permitted uninterrupted CDK4/6 inhibitor for >3 years, deferring systemic therapy changes.

The spectrum of molecular imaging radiotracers in breast cancer is rapidly expanding.^32^ Positron emission tomography using ^18^F-fluoroestradiol enables whole-body assessment of estrogen receptor expression and has emerged as a promising tool in HR+ breast cancer. Recent data suggest that ^18^F-fluoroestradiol PET/CT may identify a subset of patients who derive particular benefit from hormone therapy combined with CDK4/6 inhibitors.^33^ In parallel, ^68^Ga-labeled fibroblast activation protein inhibitor PET/CT has shown higher tumor-to-background contrast than ^18^F-FDG in breast cancer,^34^ with emerging evidence supporting its role in response assessment to neoadjuvant chemotherapy^35^ and improved tumor delineation in organs with high physiological FDG uptake.^36^ Ongoing prospective studies comparing ^18^F-FDG and ^68^Ga-labeled fibroblast activation protein inhibitor PET/CT in HR+ breast cancer (NCT06335069)^37^ and the development of FAP-targeted radiopharmaceuticals, including ^177^Lu-FAP-2286 ^38^ and ^90^Y-FAPI-46,^39^ further highlight the growing diagnostic and theranostic potential of FAP-directed strategies.

Feasibility and generalizability deserve consideration. Integrating ^18^F-FDG-PET/CT into routine practice at our center allowed precise longitudinal assessment and patient-specific timing of SBRT—either at CDK4/6 inhibitor initiation or at OPD when disease remained controlled elsewhere. Broader implementation depends on access to high-quality ^18^F-FDG-PET/CT, coordinated multidisciplinary workflows, and institutional experience. Centers without an established ^18^F-FDG-PET/CT infrastructure may require additional resources and local validation. Prospective data are needed to define the impact of ^18^F-FDG-PET/CT–adapted SBRT on long-term outcomes and to standardize operational pathways.

The main limitations of our study are inherent to its retrospective design. We lacked a structured QoL assessment, selection bias was possible, and dose prescriptions were heterogeneous. Fractionation and total dose were individualized according to lesion size, location, and proximity to organs at risk. Although such tailoring reflects real‑world multidisciplinary decision‑making, it complicates cross‑study comparisons and may confound attribution of benefit. Prospective evidence is mixed. NRG BR002, a randomized phase 2R/3 trial, tested the addition of SBRT or metastasectomy to standard systemic therapy as first-line management for oligometastatic breast cancer and did not demonstrate improvements in PFS or OS.^15^ However, the relatively short follow-up and low number of events limited the ability to detect a potential benefit of incorporating local ablative therapy with systemic CDK4/6 inhibitors.

Conversely, in a phase 2 study, SF-SBRT at 24 Gy achieved ≥92% ablation at 5 years in a large, consecutive oligometastatic cohort; however, polymetastases-free survival was only 26%, indicating that systemic progression ultimately dominates in many patients.^11^ Thus, effective systemic therapy is crucial, and the addition of other modalities should not lead to treatment cessation due to enhanced toxicity. However, it is important to acknowledge that local treatments may extend the interval before subsequent systemic therapies need to be initiated, potentially providing patients valuable time free from the higher toxicity associated with more intensive systemic treatments, such as chemotherapy.^40^

Recent meta-analyses have evaluated the safety of combining CDK4/6 inhibitors with RT, reporting pooled rates of severe hematologic and nonhematologic toxicity of approximately 29% and 3%, respectively, and a low rate of treatment discontinuation due to adverse events.^41^^,^^42^ However, most available data are derived from palliative RT settings, with patients treated using SBRT being underrepresented. In this context, the safety profile observed in our cohort is reassuring and aligns with prior reports, despite the use of higher-dose regimens, including SF-SBRT. Skeletal toxicity after SBRT, particularly vertebral compression fractures, has been reported in 10% to 30% of patients in previous series,^43^ whereas no such events were observed in our cohort. We recorded one traumatic femoral fracture; the cumulative incidence of long‑bone fracture after SBRT has been reported to be approximately 6% at 1 year, and—as in our case—most events are not associated with local failure.^44^

Treatment after CDK4/6 inhibitor progression remains challenging, with modest efficacy of current options.^45^ In our study, despite diversified approaches, including chemotherapy and mTOR inhibitors, responses to subsequent systemic treatment were poor, with a median PFS of only 6 months. Local therapy for OPD while continuing CDK4/6 inhibitors is an appealing strategy, but has been understudied. The prospective, multicenter AVATAR study (abstract) enrolled 32 patients on CDK4/6 inhibitors with OPD treated with SABR while continuing unchanged systemic therapy; 46% remained free from events at 6 months.^40^ In our series, 83% of patients remained progression-free for ≥6 months, suggesting that careful selection—potentially aided by ^18^F-FDG-PET/CT—may enhance outcomes.

Nevertheless, heterogeneity across the SBRT literature limits external validity. Many studies pool disparate histologies and treatment sites, and even within breast cancer cohorts, systemic backbones and timing vary substantially.^24^^,^^46^ Consequently, extrapolating effect sizes to contemporary HR+/HER2‑negative disease managed with CDK4/6 inhibitors is challenging. Ongoing phase 2 trials—ASPIRE (NCT03691493),^47^ CLEAR (NCT03750396),^48^ and PALATINE (NCT03870919)^49^—should clarify the role, timing, and patient selection criteria for combining SBRT with CDK4/6 inhibitors.

## Conclusions

^18^F-FDG-PET/CT–guided SBRT, particularly single-fraction, added to CDK 4/6 inhibitors, is a valuable treatment modality resulting in substantial rates of mCR and durable LC. To our knowledge, this study represents the first analysis evaluating ^18^F-FDG-PET/CT–guided SBRT in combination with CDK4/6 inhibitor–based therapy in patients with MBC.

## Disclosures

Marcin Kubeczko reports a relationship with the International Cancer Foundation that includes grant support (ESMO Clinical Unit Visit Fellowship at the Breast Unit, Champalimaud Clinical Centre); with Novartis and Gilead Sciences Inc that includes consulting fees, honoraria for lectures, support for attending meetings, and advisory board participation; with F. Hoffmann-La Roche Ltd, Merck Sharp & Dohme UK Ltd, Pfizer, AstraZeneca, and Swixx Biopharma Sp zoo that includes honoraria for lectures and support for attending meetings; and with Eli Lilly and Company that includes honoraria for lectures. Berta Sousa reports a relationship with Novartis, AstraZeneca, Pfizer, and F. Hoffmann-La Roche Ltd that includes support for attending meetings. Leonor Matos reports a relationship with Novartis and Eli Lilly and Company that includes honoraria for lectures and support for attending meetings; with Roche that includes honoraria for lectures and advisory board participation; and with Bayer that includes advisory board participation. Michał Jarząb reports a relationship with Novartis and Pfizer that includes: consulting or advisory and speaking and lecture fees; with F. Hoffmann-La Roche Ltd, Gilead Sciences Inc, Eli Lilly and Company, Teva Pharmaceutical Industries Ltd, Exact Sciences Corporation, and Mammotome that includes: speaking and lecture fees. Fatima Cardoso reports a relationship with Amgen, Astellas/Medivation, AstraZeneca, Bayer Corporation, Celgene Corporation, Daiichi Sankyo Inc, Eisai Inc, GE Oncology, Genentech Inc, Gilead Sciences Inc, GlaxoSmithKline LLC, IQVIA, MacroGenics, Medscape LLC, Merck Sharp & Dohme Corp, Merus NV, Mylan Pharmaceuticals Inc, Mundipharma, Novartis, Pfizer, Pierre Fabre SA, prIME Oncology, Roche, Sanofi, Samsung Bioepis Co Ltd, Seagen Inc, Teva Pharmaceuticals Industries Inc, and Touch Independent Medical Education Ltd that includes advisory board participation that includes advisory board participation. The other authors declare that they have no known competing financial interests or personal relationships that could have appeared to influence the work reported in this paper.

## Appendix Supplementary materials

Supplementary Table 1 Image, application 1

Supplementary Table 2 Image, application 2

## Footnotes

Supplementary material associated with this article can be found in the online version at doi:10.1016/j.adro.2026.102052.

## References

[1] Arnold M., Morgan E., Rumgay H. (2022). Current and future burden of breast cancer: Global statistics for 2020 and 2040. Breast.

[2] Cardoso F., Paluch-Shimon S., Senkus E. (2020). 5th ESO-ESMO international consensus guidelines for advanced breast cancer (ABC 5). Ann Oncol.

[3] Xu X.Q., Pan X.H., Wang T.T. (2021). Intrinsic and acquired resistance to CDK4/6 inhibitors and potential overcoming strategies. Acta Pharmacol Sin.

[4] Main S.C., Cescon D.W., Bratman S.V. (2022). Liquid biopsies to predict CDK4/6 inhibitor efficacy and resistance in breast cancer. Cancer Drug Resist.

[5] Boellaard R., Delgado-Bolton R., Oyen W.J.G. (2015). FDG PET/CT: EANM procedure guidelines for tumour imaging: Version 2.0. Eur J Nucl Med Mol Imaging.

[6] Paydary K., Seraj S.M., Zadeh M.Z. (2019). The evolving role of FDG-PET/CT in the diagnosis, staging, and treatment of breast cancer. Mol Imaging Biol.

[7] Li H., Yao L., Jin P. (2018). MRI and PET/CT for evaluation of the pathological response to neoadjuvant chemotherapy in breast cancer: A systematic review and meta-analysis. Breast.

[8] Taralli S., Lorusso M., Scolozzi V., Masiello V., Marazzi F., Calcagni M.L. (2019). Response evaluation with 18 F-FDG PET/CT in metastatic breast cancer patients treated with Palbociclib: First experience in clinical practice. Ann Nucl Med.

[9] Önner H., Eren OÖ, Körez M.K., Yilmaz F., Kara Gedik G. (2023). Comparison of prognostic value of different metabolic response criteria determined by PET/CT in patients with metastatic breast cancer under CDK 4/6 inhibitor treatment. Rev Esp Med Nucl Imagen Mol (Engl Ed).

[10] Seifert R., Küper A., Tewes M. (2021). [^18^F]-fluorodeoxyglucose positron emission tomography/CT to assess the early metabolic response in patients with hormone receptor-positive HER2-negative metastasized breast cancer treated with cyclin-dependent 4/6 kinase inhibitors. *Oncol Res Treat*. S Karger AG.

[11] Greco C., Pares O., Pimentel N. (2019). Phenotype-oriented ablation of oligometastatic cancer with single dose radiation therapy. Int J Radiat Oncol Biol Phys.

[12] Kroeze S.G.C., Pavic M., Stellamans K. (2023). Metastases-directed stereotactic body radiotherapy in combination with targeted therapy or immunotherapy: Systematic review and consensus recommendations by the EORTC–ESTRO OligoCare consortium. Lancet Oncol.

[13] Meattini I., Becherini C., Caini S. (2024). International multidisciplinary consensus on the integration of radiotherapy with new systemic treatments for breast cancer: European Society for Radiotherapy and Oncology (ESTRO)-endorsed recommendations. Lancet Oncol.

[14] Bodo S., Campagne C., Thin T.H. (2019). Single-dose radiotherapy disables tumor cell homologous recombination via ischemia/reperfusion injury. J Clin Invest.

[15] Chmura S.J., Winter K.A., Woodward W.A. (2022). NRG-BR002: A phase IIR/III trial of standard of care systemic therapy with or without stereotactic body radiotherapy (SBRT) and/or surgical resection (SR) for newly oligometastatic breast cancer (NCT02364557). J Clin Oncol.

[16] Palma D.A., Olson R., Harrow S. (2020). Stereotactic ablative radiotherapy for the comprehensive treatment of oligometastatic cancers: Long-term results of the SABR-COMET phase II randomized trial. J Clin Oncol.

[17] Ibrance. European Medicines Agency. Accessed February 15, 2023. https://www.ema.europa.eu/en/medicines/human/EPAR/ibrance

[18] Kisqali. European Medicines Agency. Accessed February 15, 2023. https://www.ema.europa.eu/en/medicines/human/EPAR/kisqali

[19] Verzenios. European Medicines Agency. Accessed February 15, 2023.https://www.ema.europa.eu/en/medicines/human/EPAR/verzenios

[20] Young H., Baum R., Cremerius U. (1999). Measurement of clinical and subclinical tumour response using [^18^F]-fluorodeoxyglucose and positron emission tomography: Review and 1999 EORTC recommendations. Eur J Cancer.

[21] Ho K.C., Fang Y.D., Chung H.W. (2016). TLG-S criteria are superior to both EORTC and PERCIST for predicting outcomes in patients with metastatic lung adenocarcinoma treated with erlotinib. Eur J Nucl Med Mol Imaging.

[22] Spring L.M., Wander S.A., Andre F., Moy B., Turner N.C., Bardia A. (2020). Cyclin-dependent kinase 4 and 6 inhibitors for hormone receptor-positive breast cancer: Past, present, and future. Lancet.

[23] Hortobagyi G.N., Stemmer S.M., Burris H.A. (2018). Updated results from MONALEESA-2, a phase III trial of first-line ribociclib plus letrozole versus placebo plus letrozole in hormone receptor-positive, HER2-negative advanced breast cancer. Ann Oncol.

[24] Lehrer E.J., Singh R., Wang M. (2021). Safety and survival rates associated with ablative stereotactic radiotherapy for patients with oligometastatic cancer: A systematic review and meta-analysis. JAMA Oncol.

[25] Baker S., Jiang W., Mou B. (2022). Progression-free survival and local control after SABR for up to 5 oligometastases: An analysis from the population-based phase 2 SABR-5 trial. Int J Radiat Oncol Biol Phys.

[26] Yang Y., Luo J., Chen X. (2020). CDK4/6 inhibitors: A novel strategy for tumor radiosensitization. J Exp Clin Cancer Res.

[27] Groheux D. (2018). Role of fludeoxyglucose in breast cancer: Treatment response. PET Clin.

[28] Constantino C.S., Oliveira F.P.M., Silva M. (2021). Are lesion features reproducible between ^18^F-FDG PET/CT images when acquired on analog or digital PET/CT scanners?. Eur Radiol.

[29] Constantino C.S., Leocádio S., Oliveira F.P.M. (2023). Evaluation of semiautomatic and deep learning–based fully automatic segmentation methods on [^18^F]FDG PET/CT images from patients with lymphoma: Influence on tumor characterization. J Digit Imaging.

[30] Vogsen M., Harbo F., Jakobsen N.M. (2023). Response monitoring in metastatic breast cancer: A prospective study comparing ^18^F-FDG PET/CT with conventional CT. J Nucl Med.

[31] Pasqualetti F., Panichi M., Sollini M. (2020). [^18^F]Fluorocholine PET/CT-guided stereotactic body radiotherapy in patients with recurrent oligometastatic prostate cancer. Eur J Nucl Med Mol Imaging.

[32] Altena R., Tzortzakakis A., Af Burén S. (2023). Current status of contemporary diagnostic radiotracers in the management of breast cancer: First steps toward theranostic applications. EJNMMI Res.

[33] Boers J, Venema CM, de Vries EFJ, et al. Molecular imaging to identify patients with metastatic breast cancer who benefit from endocrine treatment combined with cyclin-dependent kinase inhibition. 2020;126:11-20. 10.1016/j.ejca.2019.10.024 31891878

[34] Li T., Zhang J., Yan Y., Tan M., Chen Y. (2024). Applications of FAPI PET/CT in the diagnosis and treatment of breast and the most common gynecologic malignancies: A literature review. Front Oncol.

[35] Chen L., Zheng S., Chen L. (2023). ^68^Ga-labeled fibroblast activation protein inhibitor PET/CT for the early and late prediction of pathologic response to neoadjuvant chemotherapy in breast cancer patients: A prospective study. J Nucl Med.

[36] Giesel F.L., Kratochwil C., Schlittenhardt J. (2021). Head-to-head intra-individual comparison of biodistribution and tumor uptake of ^68^Ga-FAPI and ^18^F-FDG PET/CT in cancer patients. Eur J Nucl Med Mol Imaging.

[37] 18F-FDG versus 68Ga-FAPI-46 as PET tracer in ER-positive breast cancer. ClinicalTrials.gov identifier: NCT06335069. Updated September 24, 2025. https://clinicaltrials.gov/study/NCT06335069

[38] Baum R.P., Schuchardt C., Singh A. (2022). Feasibility, biodistribution, and preliminary dosimetry in peptide-targeted radionuclide therapy of diverse adenocarcinomas using ^177^Lu-FAP-2286: First-in-humans results. J Nucl Med.

[39] Rathke H., Fuxius S., Giesel F.L. (2021). Two tumors, one target: Preliminary experience with ^90^Y-FAPI therapy in a patient with metastasized breast and colorectal cancer. Clin Nucl Med.

[40] David S.P., Siva S., Bressel M. (2023). Stereotactic ablative body radiotherapy (SABR) for oligoprogressive ER-positive breast cancer (AVATAR): A phase II prospective multicenter trial. Int J Radiat Oncol.

[41] Becherini C., Visani L., Caini S. (2023). Safety profile of cyclin-dependent kinase (CDK) 4/6 inhibitors with concurrent radiation therapy: A systematic review and meta-analysis. Cancer Treat Rev.

[42] Kubeczko M., Jarząb M., Gabryś D., Krzywon A., Cortez A.J., Xu A.J. (2023). Safety and feasibility of CDK4/6 inhibitors treatment combined with radiotherapy in patients with HR-positive/HER2-negative breast cancer. A systematic review and meta-analysis. Radiother Oncol.

[43] Guckenberger M., Dahele M., Ong W.L., Sahgal A. (2023). Stereotactic body radiation therapy for spinal metastases: Benefits and limitations. Semin Radiat Oncol.

[44] Madani I., Sahgal A., Erler D. (2022). Stereotactic body radiation therapy for metastases in long bones. Int J Radiat Oncol Biol Phys.

[45] Mittal A., Molto Valiente C., Tamimi F. (2023). Filling the gap after CDK4/6 inhibitors: Novel endocrine and biologic treatment options for metastatic hormone receptor positive breast cancer. Cancers (Basel).

[46] Gennari A., André F., Barrios C.H. (2021). ESMO Clinical Practice Guideline for the diagnosis, staging and treatment of patients with metastatic breast cancer. Ann Oncol.

[47] Radiation therapy, palbociclib, and hormone therapy in treating breast cancer patients with bone metastasis (ASPIRE). ClinicalTrials.gov identifier: NCT03691493. Updated February 17, 2025. https://clinicaltrials.gov/study/NCT03691493

[48] Local treatment in ER-positive/HER2-negative oligo-metastatic breast cancer (CLEAR). ClinicalTrials.gov identifier: NCT03750396. Updated February 15, 2019.https://clinicaltrials.gov/study/NCT03750396

[49] Locoregional treatment and palbociclib in de novo, treatment naive, stage IV ER+, HER2- breast cancer patients (PALATINE). ClinicalTrials.gov identifier: NCT03870919. Updated September 19, 2024. https://clinicaltrials.gov/study/NCT03870919

