# Supplementary material for: 18F-Fluorodeoxyglucose–Positron Emission Tomography/Computed Tomography Guided Stereotactic Body Radiation Therapy in Advanced Breast Cancer Patients Treated WithCyclin-Dependent Kinase 4/6 Inhibitors
Source: Adv Radiat Oncol. 2026 Apr 7;11(7):102052. doi: 10.1016/j.adro.2026.102052 (PMC13234201; doi:10.1016/j.adro.2026.102052)
Supplement: Supplementary Table 1 [file mmc1.docx]

**Supplementary Table 1.** Details on SBRT timing during CDK4/6 inhibitors treatment in patients irradiated concomitantly at the beginning of CDK4/6 inhibitors or due to oligoprogression

| Pt  Nb | Age | Treated site | Dt/Dfx | CDK4/6 inhibitors | CDK4/6 inhibitors suspension | Nb of days with SBRT while taking CDK4/6 inhibitors | Nb of days with SBRT including CDK4/6i inhibitors t1/2 | % of SBRT while on CDK4/6 inhibitors* |
| --- | --- | --- | --- | --- | --- | --- | --- | --- |
| SBRT performed at the beginning of CDK4/6 inhibitors treatment | | | | | | | | |
| 1 | 57 | T8-9 | 25/5 | abemaciclib | susp. on SBRT D2 | 1 | 5 | 100 |
| 11 | 57 | Sacrum+Ilia (R+L) & Femur R | 25/5 | ribocilib | no | 5 | 5 | 100 |
| 14 | 61 | T 6-8 &  Rib 10 L | 25/5 | ribociclib | no | 5 | 5 | 100 |
| 16 | 48 | T 9 & Skull | 24/24 | palbociclib | no | 1 | 1 | 100 |
| 17 | 74 | Sacrum-  Ilium R | 30/6 | palbociclib | susp. on SBRT D1 | 0 | 5 | 100 |
| 23 | 59 | T11 & Sacrum-  Ilium L | 35/7 | palbociclib | susp. on SBRT D1 | 0 | 5 | 100 |
| 24 | 58 | Sacrum | 25/5 | palbociclib | no (SBRT finished on CDK C1D1) | 1 | 1 | 20 |
| 28 | 61 | Rib 12 R & T7 | 36/12 | palbociclib | susp. 7d before SBRT | 0 | 0 | 0 |
| SBRT performed due to oligoprogression | | | | | | | | |
| 2 | 54 | Ilium L (2 lesions) & L5 | 30/6 | palbociclib | during week off (SBRT started on D25 of CDK cycle) | 0 | 2 | 40 |
| 8 | 64 | Sacrum | 24/24 | ribociclib | no | 1 | 1 | 100 |
| 8 | 64 | T11 | 35/7 | ribociclib | no | 5 | 5 | 100 |
| 8 | 64 | Ilium L | 24/24 | ribociclib | no | 1 | 1 | 100 |
| 9 | 48 | Mediastinal LNs  (2 lesions) | 35/7 | ribociclib | susp. on SBRT D1 | 0 | 5 | 100 |
| 10 | 57 | L1 & Th4 | 20/20 | ribociclib | during week off, (SBRT on D25 of CDK cycle | 0 | 1 | 100 |
| 13 | 64 | T8 & Ilium R | 16/16 | ribociclib | susp. due to G3 neutr. | 0 | 0 | 0 |
| 15 | 54 | Liver metastasis | 45/15 | palbociclib | during week off, on D24, D26, and D28 of CDK cycle | 0 during 7 days off; days 26-28 | 2 | 66.7 |
| 17 | 74 | L4 | 14/14 | palbociclib | no | 1 | 1 | 100 |
| 18 | 40 | L4 | 21/21 | palbociclib | no | 1 | 1 | 100 |
| 22 | 83 | T11 | 24/24 | palbociclib | no | 1 | 1 | 100 |
| 23 | 59 | T6 | 35/7 | palbociclib | no | 5 | 5 | 100 |
| 27 | 66 | L5 & Ilium L | 24/24 | ribociclib | susp. on SBRT D1 | 0 | 1 | 100 |
| 29 | 77 | Sacrum+Ilium R (3 lesions) | 16/16 | palbociclib | no | 1 | 1 | 100 |

*including CDK4/6 inhibitors t1/2

Abbreviations: Pt Nb – Patient number; Dt/Dfx – total dose/dose per fraction; LC mth – local control in months, RTint(days) – interval between SBRT completion and CDK4/6 inhibitors commencement, in days; C – cervical spine; T – thoracic spine, L – lumbar spine; LNs – lymph nodes; R – right; L- left; susp. – suspension; C1D1 – day 1 of cycle 1 of CDK4/6 inhibitor treatment
