# Supplementary material for: 18F-Fluorodeoxyglucose–Positron Emission Tomography/Computed Tomography Guided Stereotactic Body Radiation Therapy in Advanced Breast Cancer Patients Treated WithCyclin-Dependent Kinase 4/6 Inhibitors
Source: Adv Radiat Oncol. 2026 Apr 7;11(7):102052. doi: 10.1016/j.adro.2026.102052 (PMC13234201; doi:10.1016/j.adro.2026.102052)
Supplement: Supplementary Table 2 [file mmc2.docx]

**Supplementary Table 2.** Irradiated volumes.

| Pt Nb | Age | Treated Site | CTV(cm^3^) | PTV(cm^3^) | Dose prescribed to PTV Dt/Dfx | CTV boost (cm^3^) | PTV boost (cm^3^) | Dose prescribed to boost volume Dt/Dfx |
| --- | --- | --- | --- | --- | --- | --- | --- | --- |
| 1 | 57 | Sacrum+Ilium R | 652.7 | 1021 | 25/5 | 0 | 0 | 0 |
| 1 | 57 | T8-9 | 53.5 | 93.9 | 25/5 | 0 | 0 | 0 |
| 2 | 54 | T6 | 10.8 | 22.1 | 16/16 | 1.7 | 2.8 | 24/24 |
| 2 | 54 | Ilium R | 29.3 | 53.6 | 16/16 | 0 | 0 | 0 |
| 2 | 54 | Ilium R | 1.7 | 5.1 | 24/24 | 0 | 0 | 0 |
| 2 | 54 | Ilium R | 0.3 | 1.7 | 24/24 | 0 | 0 | 0 |
| 2 | 54 | Ilium L | 0.6 | 2.5 | 24/24 | 0 | 0 | 0 |
| 2 | 54 | Scapula L | 6.4 | 16.3 | 24/24 | 0 | 0 | 0 |
| 2 | 54 | Ilium L | 2 | 5.9 | 30/6 | 0 | 0 | 0 |
| 2 | 54 | Ilium L | 1.7 | 5.3 | 30/6 | 0 | 0 | 0 |
| 2 | 54 | Ilium L | 0.8 | 3.1 | 30/6 | 0 | 0 | 0 |
| 2 | 54 | L5 | 57.5 | 100.7 | 25/5 | 21.2 | 39.5 | 30/6 |
| 3 | 58 | Supraclavicular LN L | 7.5 | 18.9 | 25/5 | NR | 4.3 | 35/7 |
| 4 | 68 | C2 | 7.9 | 20.4 | 25/5 | 0 | 0 | 0 |
| 4 | 68 | L3 | 37.8 | 63.7 | 25/5 | 0 | 0 | 0 |
| 4 | 68 | L5 | 49.9 | 85 | 25/5 | 0 | 0 | 0 |
| 5 | 68 | Ischium L | 4.8 | 12.3 | 24/24 | 0 | 0 | 0 |
| 5 | 68 | Sacrum | 78.9 | 126 | 21/21 | 0 | 0 | 0 |
| 6 | 86 | Ilium R + Sacrum | 455.7 | 595.1 | 25/5 | 0 | 0 | 0 |
| 7 | 75 | Mediastinal LNs  (2 lesions, one CTV and PTV) | 1.3 | 5.8 | 21/21 | 0 | 0 | 0 |
| 8 | 64 | Sternum | 78.3 | 120.3 | 35/7 | 0 | 0 | 0 |
| 8 | 64 | Sacrum | 1.1 | 4 | 24/24 | 0 | 0 | 0 |
| 8 | 64 | T11 | 1.3 | 4.6 | 35/7 | 0 | 0 | 0 |
| 8 | 64 | Ilium L | 1.6 | 4.9 | 24/24 | 0 | 0 | 0 |
| 9 | 48 | Humerus R | 2.6 | 8.6 | 21/21 | 0 | 0 | 0 |
| 9 | 48 | Mediastinal LN  (2 lesions, 2 PTVs) | 0.4 | 4 | 30/6 | NR | 0.4 | 35/7 |
| 9 | 48 | Mediastinal LN | 3.3 | 10.9 | 30/6 | NR | 3.3 | 35/7 |
| 10 | 57 | Ilium L | 384.2 | 772.4 | 25/5 | 0 | 0 | 0 |
| 10 | 57 | L1 | 24.7 | 43.6 | 16/16 | NR | 24.7 | 20/20 |
| 10 | 57 | T4 | 9.7 | 21.3 | 16/16 | NR | 9.7 | 20/20 |
| 11 | 57 | Femur R | 64.6 | 104.3 | 25/5 | 0 | 0 | 0 |
| 11 | 57 | Sacrum + Ilia (R+L) | 229 | 372.9 | 25/5 | 0 | 0 | 0 |
| 12 | 63 | C3 | 7.1 | 18.1 | 25/5 | 0 | 0 | 0 |
| 12 | 63 | Ilium R | 38 | 79.9 | 35/7 | 0 | 0 | 0 |
| 13 | 64 | Femur R | 134.1 | 196.2 | 30/6 | 0 | 0 | 0 |
| 13 | 64 | T8 | 13.9 | 27.9 | 14/14 | NR | 6.1 | 16/16 |
| 13 | 64 | Ilium R | 3.8 | 9.5 | 16/16 | 0 | 0 | 0 |
| 14 | 61 | T 6-8 | 114.5 | 177.7 | 25/5 | 0 | 0 | 0 |
| 14 | 61 | Rib 10 L | 19.2 | 39.8 | 25/5 | 0 | 0 | 0 |
| 15 | 54 | Liver metastasis | 7.7 | 31.1 | 37.5/12.5 | NR | 7.7 | 45/15 |
| 16 | 48 | T 9 | 2.0 | 5.8 | 24/24 | 0 | 0 | 0 |
| 16 | 48 | Skull | 3.5 | 14.9 | 22/22 | 0 | 0 | 0 |
| 17 | 74 | Sacrum-Ilium R | 87.7 | 133.2 | 30/6 | 0 | 0 | 0 |
| 17 | 74 | L4 | 1.3 | 4.5 | 14/14 | 0 | 0 | 0 |
| 18 | 40 | L4 | 14.1 | 26.2 | 21/21 | 0 | 0 | 0 |
| 19 | 48 | L3 | 1.9 | 5.8 | 24/24 | 0 | 0 | 0 |
| 19 | 48 | L4 | 1.4 | 4.7 | 24/24 | 0 | 0 | 0 |
| 20 | 85 | L5 | 37.7 | 68.0 | 35/7 | 0 | 0 | 0 |
| 21 | 46 | Lumbosacral | 550.5 | 912.6 | 25/5 | 0 | 0 | 0 |
| 22 | 83 | T11 | 24.6 | 45.5 | 16/16 | 2.1 | 6.7 | 24/24 |
| 23 | 59 | T11 | 35.0 | 61.9 | 25/5 | 6.8 | 13.8 | 35/7 |
| 23 | 59 | Sacrum-Ilium L | 115.4 | 170.0 | 35/7 | 0 | 0 | 0 |
| 23 | 59 | T6 | 2.8 | 7.7 | 30/6 | NR | 2.8 | 35/7 |
| 24 | 58 | Sacrum | 58.1 | 129.1 | 25/5 | 0 | 0 | 0 |
| 25 | 48 | Internal Mammary LNs  (4 LNs, 4 PTVs) | 7.5 | 32.1 | 35/7 | 0 | 0 | 0 |
| 25 | 48 | Internal Mammary LNs | 0.3 | 2.9 | 35/7 | 0 | 0 | 0 |
| 25 | 48 | Internal Mammary LNs | 0.5 | 3.7 | 35/7 | 0 | 0 | 0 |
| 25 | 48 | Internal Mammary LNs | 0.6 | 4.6 | 35/7 | 0 | 0 | 0 |
| 25 | 48 | Rib 9 R | 5.8 | 21.8 | 24/24 | 0 | 0 | 0 |
| 26 | 52 | T 5-9 + ribs 6-9 | 244.8 | 376.1 | 25/5 | 0 | 0 | 0 |
| 26 | 52 | Ilium R | 86.0 | 137.0 | 25/5 | 0 | 0 | 0 |
| 27 | 66 | L5 | 33.6 | 57.6 | 16/16 | 17 | 31.8 | 24/24 |
| 27 | 66 | Iliac L | 25.0 | 43.8 | 24/24 | 0 | 0 | 0 |
| 28 | 61 | Rib 12 R | 5.1 | 16.5 | 36/12 | 0 | 0 | 0 |
| 28 | 61 | T7 | 12.2 | 25.0 | 30/10 | 6.9 | 15 | 36/12 |
| 29 | 77 | T12 | 21.4 | 33.4 | 12/12 | NR | 9.4 | 18/18 |
| 29 | 77 | L2 | 21.5 | 31.8 | 12/12 | NR | 2.2 | 18/18 |
| 29 | 77 | Sacrum | 5.4 | 10.9 | 16/16 | 0 | 0 | 0 |
| 29 | 77 | Sacrum | 30.8 | 77 | 12/12 | 0 | 0 | 0 |
| 29 | 77 | Ilium R | 39.4 | 86.3 | 16/16 | 0 | 0 | 0 |

Abbreviations: Pt Nb – Patient number; Dt/Dfx – total dose/dose per fraction; CTV – clinical target volume; PTV – planning target volume; C – cervical spine; T – thoracic spine, L – lumbar spine; LNs – lymph nodes; R – right; L- left;
